# Antioxidant profiling and quality assessment of *Lithocarpus polystachyus* sweet tea using LC-ECD and LC-MS/MS

**DOI:** 10.1038/s41598-025-97875-7

**Published:** 2025-04-16

**Authors:** Jinfen Zheng, Wei Pan, Miaoxin Liu, Yan Yu, Youyin Zhao, Ke Su, Jiao Du, Jie Li, Yaping Zhou, Rongxiang Chen

**Affiliations:** 1 Department of Analytical Chemistry, School of Pharmacy, Zunyi Medical University, Zunyi, 563000 China; 2 Analysis and Testing Center, School of Basic Medical Sciences, Zunyi Medical University, Zunyi, 563000 China

**Keywords:** *Lithocarpus polystachyus*, Sweet tea, LC-ECD, Chemical fingerprinting, Antioxidant activity, Phenolic components, Mass spectrometry, Isolation, separation and purification, Mass spectrometry, Natural products, Secondary metabolism

## Abstract

This study used liquid chromatography-electrochemical detection (LC-ECD) combined with liquid chromatography-tandem mass spectrometry (LC-MS/MS) to screen and characterize the antioxidant components of sweet tea (*Lithocarpus polystachyus*), and to evaluate the quality of the samples from different origins. First, the total phenolic and flavonoid content of sweet tea, as well as the in vitro antioxidant activity, including ABTS and DPPH radical scavenging capacities and ferric reducing ability, were determined. Samples from different origins demonstrated potent antioxidant activity, with correlation coefficients exceeding 0.8 between total phenolic content and antioxidant activities. Then, the fingerprints of sweet tea were generated by LC-ECD and 22 common peaks were identified by LC-MS/MS, with most of them being phloretin derivatives. The clustering heat map revealed differences in the content of sweet tea from various regions, with major compounds identified as trilobatin, phlorizin, 3-hydroxyphlorizin, and its isomer. Furthermore, grey relational analysis confirmed a strong association between different compounds (protocatechuic acid, epicatechin, 3-hydroxyphlorizin, isoquercitrin, and trilobatin) and antioxidant activity, with trilobatin showing the highest contribution, reaching a correlation of 0.9. Finally, 11 phenolic compounds were quantitatively determined. This study screened and identified the major antioxidant components of sweet tea using LC-ECD and LC-MS/MS, providing guidance for its quality control.

**Supplementary Information:**

The online version contains supplementary material available at 10.1038/s41598-025-97875-7.

## Introduction

*Lithocarpus polystachyus*, belonging to the Fagaceae family and distributed in the southwestern and southern provinces of China, Vietnam, India, and Thailand^1^, is renowned for its sweet-tasting leaves traditionally consumed as “sweet tea” for its cooling and refreshing properties^2^. It is 300 times sweeter than sucrose^3^, and was certified as a novel food ingredient in China in 2017^4^. As a unique resource with combined functions of tea, sweetener, and herbal medicine, sweet tea serves not only as an ideal natural sweetener^4^, but also as a research hotspot for metabolic disease prevention and treatment due to its multi-target pharmacological activity.

Studies have revealed that sweet tea contains a variety of bioactive compounds such as flavonoids, polyphenolics, and triterpenoids^5,6^, which possess multifunctional effects including antioxidant, hypolipidemic, antimicrobial, anti-allergic, anti-inflammatory, antidiabetic, hypotensive, anti-obesity, and antitumor activities^4,7–10^. Among them, phenolic compounds also have the effect of enhancing capillary vessels, inhibiting and delaying arteriosclerosis, and preventing coronary heart disease^11^. The phenolic compounds in sweet tea primarily include dihydrochalcones (e.g., phlorizin, trilobatin) and flavonoids (e.g., quercetin, quercitrin)^2^. Among these, dihydrochalcones are key bioactive ingredients that regulate glucose and lipid metabolism disorders, alleviate allergic asthma, and exhibit anticariogenic properties^4^. Additionally, they demonstrate radical scavenging and anti-aging effects^4^. Phlorizin and 3-hydroxyphlorizin are well absorbed in the Caco-2 cell monolayer model^12^. Moreover, trilobatin has been demonstrated to have a significant inhibitory effect on α-glucosidase similar to acarbose, and is capable of scavenging DPPH radicals more strongly than rutin^6^, helping prevent and treat type 2 diabetes^13^. Phlorizin has been shown to have hypoglycemic and hypolipidemic effects^14^, and can improve hepatic insulin resistance by inhibiting oxidative stress and lipid accumulation in IR-HepG2 cells^15^, making it a potential sugar substitute for diabetic patients. Recent research shows that sweet tea extract not only attenuates mitochondrial oxidative stress and neuroinflammation^16^, but also modulates intestinal microbiota and prevents ulcerative colitis in mice^17^. The toxicological evaluation showed that sweet tea aqueous extract at high doses only induced reversible liver enzyme fluctuations without genotoxicity^18^. Numerous studies have indicated that the medicinal value of sweet tea is closely related to its high content of phenolics^19^.

Despite these advances, existing research mainly focuses on the quantitatively limited components, such as phlorizin, trilobatin, and other components^20,21^, leaving the comprehensive antioxidant profile and its bioactivity correlation underexplored. Conventional methods like HPLC-UV lack sensitivity for oxidizable phenolics^22,23^, while isolated component analyses fail to capture synergistic effects. Electrochemical detection (ECD), which has excellent sensitivity and selectivity, is a commonly used method in HPLC that supplements ultraviolet and fluorescence detection. Phenolic compounds in sweet tea, containing phenolic hydroxyl groups, are prone to oxidation, enabling ECD to selectively screen redox-active moieties^24^. It plays an important role in quality control for foods, medicines, and other fields^25^. When combined with LC-MS/MS for structural analysis and multivariate statistical analysis, this method can be used to systematically evaluate the quality marker and active linker compounds.

This study combines LC-ECD and LC-MS/MS techniques for screening active ingredients and advances the field from fragment component analysis to overall quality activity assessment in sweet tea research. Our results provide insights into industrial applications, including functional food development and pharmacological exploration, and apply innovative LC-ECD/LC-MS/MS techniques for qualitative and quantitative analysis of common peaks.

## Materials and reagents

### Reagents and samples

Protocatechuic acid, catechin, gallic acid, and epicatechin were purchased from Innochem Science and Technology Co., Ltd. (Beijing, China); quercetin, *p*-coumaric acid, isoquercitrin, phlorizin, trilobatin, 3-hydroxyphlorizin, phloretin, and rutin were purchased from Pufide Biotechnology Co., Ltd. (Chengdu, China); the purity of all references was ≥ 97%. Ammonium formate, formic acid, acetonitrile, acetic acid, and methanol were of HPLC grade and purchased from Innochem Technology Co., Ltd. The water used in the experiment was ultrapure and prepared by an Elix Advantage system (Merck Millipore).

Twenty-three batches of sweet tea (*Lithocarpus polystachyus*) grown in the natural environment were collected from seven different provinces across China, namely Yunnan, Guizhou, Sichuan, Jiangxi, Anhui, Hunan, and Guangxi. The plant material was authenticated by Dr. Meng Lingjie from Zunyi Medical University (Zunyi, Guizhou Province, China). The specimens were stored in the GA2 sample storage room of the Department of Analytical Chemistry, Zunyi Medical University (Zunyi, Guizhou, China) at 20 ± 5 °C, 35-75% relative humidity, and protected from light and stored in airtight containers. The picking period for sweet tea was from April to July 2022. For detailed information, please refer to Table 1.

**Table 1:** Collection time and system location of 23 batches of samples studied.

Sample	Picking time	Origin	Coordinates	Soil	pH	Temperature
S1	2022.4	Guangnan, Yunnan	24°20′N, 105°30′E	Red/Yellow ~ red soil	4.0 ~ 5.5	17.2 ℃
S2	2022.4	Guangnan, Yunnan	24°20′N, 105°30′E	Red/Yellow ~ red soil	4.0 ~ 5.5	17.2 ℃
S3	2022.4	Guangnan, Yunnan	24°20′N, 105°30′E	Red/Yellow ~ red soil	4.0 ~ 5.5	17.2 ℃
S4	2022.4	Guangnan, Yunnan	24°20′N, 105°30′E	Red/Yellow ~ red soil	4.0 ~ 5.5	17.2 ℃
S5	2022.4	Jian, Jiangxi	27°20′N, 114°54′E	Red soil	4.5 ~ 6.5	23.5 ℃
S6	2022.6	Luan, Anhui	31°23′N, 116°19′E	Yellow/brown soil	5.0 ~ 6.5	28.4 ℃
S7	2022.4	Pingxiang, Jiangxi	27°40′N, 113°53′E	Red soil	4.5 ~ 6.0	17.3 ℃
S8	2022.6	Liangping, Sichuan	30°25′N, 107°24′E	Purple soil	5.3 ~ 8.5	18.7 ℃
S9	2022.4	Liangping, Sichuan	30°25′N, 107°24′E	Purple soil	5.3 ~ 8.5	18.7 ℃
S10	2022.5	Pingxiang, Jiangxi	27°40′N, 113°53′E	Purple soil	4.5 ~ 6.0	18.7 ℃
S11	2022.4	Liangping, Sichuan	30°25′N, 107°24′E	Purple soil	5.3 ~ 8.5	18.7 ℃
S12	2022.7	Pingxiang, Jiangxi	27°40′N, 113°53′E	Red soil	4.5 ~ 6.0	17.3 ℃
S13	2022.4	Shangrao, Jiangxi	28°27′N, 117°57′E	Red soil	4.5 ~ 5.2	21.4 ℃
S14	2022.4	Pingxiang, Jiangxi	27°40′N, 113°53′E	Red soil	4.5 ~ 6.0	17.3 ℃
S15	2022.6	Changsha, Hunan	28°14′N, 112°56′E	Red soil	4.5 ~ 6.0	28.4 ℃
S16	2022.7	Bijie, Guizhou	26°21′N, 103°36′E	Yellow/red soil	4.7 ~ 6.1	24.0 ℃
S17	2022.4	Zunyi, Guizhou	27°13′N, 106°17′E	Yellow/red soil	4.5 ~ 6.5	20.0 ℃
S18	2022.4	Zunyi, Guizhou	27°13′N, 106°17′E	Yellow/red soil	4.5 ~ 6.5	20.0 ℃
S19	2022.4	Bijie, Guizhou	26°21′N, 103°36′E	Yellow/red soil	4.7 ~ 6.1	24.0 ℃
S20	2022.4	Jian, Jiangxi	27°20′N, 114°54′E	Red soil	4.5 ~ 6.5	23.5 ℃
S21	2022.5	Jian, Jiangxi	27°20′N, 114°54′E	Red soil	4.5 ~ 6.5	23.5 ℃
S22	2022.4	Hechi, Guangxi	24°08′N, 107°15′E	Red/yellow soil	4.7 ~ 6.5	22.0 ℃
S23	2022.4	Hechi, Guangxi	24°08′N, 107°15′E	Red/yellow soil	4.7 ~ 6.5	22.0 ℃

### Preparation of sample extracts

According to the literature, drying at 40 °C can minimize the degradation of thermosensitive components such as phenolics and flavonoids^26^. An 80% aqueous methanol solution, being a moderately polar solvent, has a significantly better extraction efficiency for phenolics from plants compared to pure water or pure organic solvents^27^. Moreover, ultrasound at 40 kHz can destroy cell walls via the low-frequency cavitation effect, and an extraction time of 30 min can achieve optimal efficiency and stability^28^.

Therefore, the samples were oven-dried (GZX-9070 MBE, Boxun Industrial Co., Ltd, China) at 40 ℃, crushed, and passed through a 60-mesh sieve. Subsequently, 1 g of the powder was weighed and added to 30 mL of 80% aqueous methanol. The sample was then subjected to ultrasonic (SB-5200DT, Xinzhi biological Co., Ltd, China) extraction for 30 min at 40 kHz. The mixture was centrifuged at 9150 × g for 5 min using a centrifuge (Microfuge-20, Beckman, Germany). The supernatant was collected and filtered through a 0.22 μm microporous filter membrane. The filtered solution was immediately subjected to LC-ECD analysis. The supernatant was dispensed into freezing tubes and stored at -20 °C. The subsequent activity assay should be completed within 3 days.

### Preparation of standard solutions

Appropriate amounts of protocatechuic acid, catechin, phlorizin, trilobatin, 3-hydroxyphlorizin, phloretin, *p*-coumaric acid, isoquercitrin, epicatechin, quercetin, and quercitrin were accurately weighed and mixed with 80% aqueous methanol to prepare a standard solution with a concentration of 2 mg/mL and stored at -20 ℃. Before use, the solution was diluted with 80% aqueous methanol to the required concentration.

## Methods

### Determination of total phenolic content (TPC)

The TPC was determined following a reference method^29,30^. The sample was diluted 180 times using 80% aqueous methanol. Then, 250 µL of the diluted sample was mixed with an equal volume of Folin-Ciocalteu reagent (0.25 mol/L). After reacting for 3 min, 500 µL of 15% Na_2_CO_3_ solution was added, and the reaction mixture was kept in the dark for 30 min. The absorbance was measured at 760 nm using an IMARK microplate reader (Bio-Rad, USA). The TPC was calculated and expressed as gallic acid equivalent per gram (mg GAE/g).

### Determination of total flavonoid content (TFC)

The TFC was determined according to the reference method^31^. The sample solution was diluted 24 times with 80% aqueous methanol. Next, 1500 µL of the diluted sample was mixed with 250 µL of 10% NaNO_2_ solution. Then, 250 µL of 10% Al(NO_3_)_3_ solution was added, followed by 2 mL of 4% NaOH solution. After thorough mixing, the mixture was left to stand for 15 min. The absorbance was measured at 510 nm. The TFC was calculated and expressed as rutin equivalent per gram (mg RE/g).

### Evaluation of antioxidant activity

#### DPPH radical scavenging capacity assay

The DPPH radical scavenging capacity assay was carried out by employing the method of reference with slight modifications^32,33^. The sample was diluted 80 times with 80% aqueous methanol. Subsequently, 200 µL of the diluted sample was mixed with 400 µL of DPPH solution as the sample group. For the preparation of the blank group, 80% aqueous methanol was used in place of the sample. After incubating in the dark for 10 min, the absorbance (A) was measured at 517 nm. The absorbance of the sample group (A_s_) and the blank group (A_0_) was recorded. The scavenging rate R was calculated according to the formula: R = [(A_0_ – A_s_) / A_0_] × 100%.

#### ABTS radical scavenging capacity assay

The ABTS radical scavenging capacity assay was conducted by utilizing the method of reference with slight modifications^34^. The ABTS reagent was prepared and diluted to achieve an absorbance of 0.8 ± 0.02 at 734 nm to obtain the ABTS working solution. After diluting the sample 400 times with 80% aqueous methanol, 100 µL of the sample was mixed with 200 µL of ABTS reagent to form the sample group. In contrast, 80% aqueous methanol was used as the blank group instead of the sample. After subjecting the sample to dark reaction at room temperature for 30 min, the absorbance was measured at 734 nm. The absorbances in the sample group and the blank group were respectively expressed as A_s_ and A_0_, respectively. The scavenging rate R was calculated using the formula: R = [(A_0_ – A_s_) / A_0_] × 100%.

#### Ferric reducing antioxidant power (FRAP) assay

The TPTZ working solution was prepared and FRAP was determined following the method of reference^35,36^. The sample was diluted 80 times with 80% aqueous methanol. Then, 100 µL of the diluted sample was mixed with 300 µL of TPTZ working solution and left to stand at room temperature for 5 min. The absorbance was measured at 593 nm. The absorption values of different concentrations of Trolox were measured under the same conditions, and the standard curve was plotted. The FRAP results were expressed as the equivalent amount of Trolox per gram (mg TE/g).

### LC-ECD conditions

The Ultimate 3000 bio-RS system (Thermo Fisher Scientific, USA) equipped with an ECD-3000RS electrochemical detector and Chromeleon 7.2 workstation was used for LC-ECD analysis. The stationary phase was an Xbridge BEH shield RP18 column (3.0 mm × 150 mm, 2.5 μm) (Waters, USA). The mobile phase consisted of 25 mmol/L ammonium formate-acetonitrile (A) and ammonium formate-water with a pH of 2.9 (B). The elution gradient was as follows: 0 to 5 min, 2.5 − 10% A; 5–7 min, 10 − 15% A; 7–20 min, 15 − 19.5% A; 20–52 min, 19.5 − 32% A; 52–58 min, 32 − 53% A; 58–60 min, 53 − 95% A. The flow rate was 0.6 mL/min, the injection volume was 1 µL, the column temperature was 40 °C, and the sample tray temperature was 12 °C. The detection potential was set at 700 mV. Under the above conditions, mixed standard solutions at various concentrations were injected, and the peak area was plotted against the concentration to construct a standard curve. An appropriate amount of the standard was added to the sample at a known concentration and the recovery was calculated to evaluate the accuracy.

### Identification of the compounds by LC-MS/MS

The e2695 HPLC equipped with an Xbridge BEH Shield RP18 column (10 × 150 mm, 5 μm) (Waters, USA) was employed for the preparation of fractions containing major compounds. The mobile phase was methanol and 0.1% aqueous formic acid. Various fractions were collected, and the compounds were identified using an I-Class-TQ-S mass spectrometer (Waters, USA) in negative mode, with Scan Mode (m/z 100–900) and Product Ion Analysis to obtain precursor and fragment ions.

### Statistical analysis

Microsoft Excel 2021 software (Redmond, WA, USA) was employed for data processing. Data were presented as mean ± SD (standard deviation) (*n* = 3). Analysis of variance (ANOVA), Tukey’s HSD test, and gray correlation analysis (GRA) plots were conducted using SPSS Statistics 27 software (Chicago, Illinois, USA). Clustered heat map and Pearson’s correlation plots were generated using Origin 2021 (Hampden, MA, USA).

## Results and discussion

### TPC, TFC and antioxidant activity of sweet tea

The TPC, TFC, and in vitro antioxidant activities, including DPPH and ABTS radical scavenging capacities as well as FRAP of 23 batches of sweet tea were determined. Statistical differences were expressed based on one-way ANOVA test (*P* < 0.01), the results are presented in Table 2. It is evident that the TPC and TFC of different samples vary between 15.29 ~ 107.27 mg/g and 10.08 ~ 97.28 mg/g, respectively. The radical scavenging capacities of DPPH and ABTS were between 30.24%~83.98% and 45.95%~91.88%, respectively, while the FRAP values ranged from 30.54 to 65.17 mg/g. There were differences in the antioxidant activity between different batches of sweet tea. The antioxidant experimental results for S16 and S17 were relatively good and similar to each other. ANOVA analysis indicated statistically significant differences between the groups (*P* < 0.05). A subsequent Tukey’s HSD test further revealed significant differences in the DPPH, ABTS, and FRAP metrics (*P* < 0.05), leading to the conclusion that the antioxidant capacity of S17 was statistically significantly higher than that of S16. In contrast, the results for S3 and S4 were relatively poor and close. ANOVA showed no significant difference between them (*P* > 0.05), suggesting that both were less effective in terms of antioxidant activity.

**Table 2:** TPC, TFC and antioxidant activity capacities of sweet tea. TPC total phenolic content, TFC total flavonoid content, FRAP ferric reducing antioxidant power. The value is the mean ± sd.

Sample	TPC (mg GAE/g)	TFC (mg RE/g)	FRAP (mg TE/g)	DPPH Radical scavenging capacity (%)	ABTS Radical scavenging capacity (%)
S1	40.95 ± 0.09	23.01 ± 0.23	35.38 ± 0.21	49.94 ± 0.06	55.36 ± 0.08
S2	15.47 ± 0.13	10.74 ± 0.22	30.83 ± 0.13	35.09 ± 0.09	51.23 ± 0.09
S3	15.29 ± 0.15	10.08 ± 0.10	30.54 ± 0.12	31.06 ± 0.04	45.95 ± 0.13
S4	15.82 ± 0.14	20.70 ± 0.33	30.89 ± 0.23	30.24 ± 0.10	50.15 ± 0.01
S5	78.41 ± 0.18	47.35 ± 0.10	39.38 ± 0.07	66.96 ± 0.07	83.26 ± 0.09
S6	88.18 ± 0.07	65.10 ± 0.35	59.74 ± 0.08	75.49 ± 0.39	90.97 ± 0.06
S7	85.90 ± 1.33	68.70 ± 0.25	58.03 ± 0.08	75.76 ± 0.43	83.60 ± 0.33
S8	68.82 ± 0.03	43.50 ± 0.34	38.53 ± 0.02	65.51 ± 0.28	76.42 ± 0.32
S9	53.38 ± 0.06	53.67 ± 0.14	41.21 ± 0.07	54.87 ± 0.15	65.42 ± 0.14
S10	70.84 ± 0.07	75.84 ± 0.70	48.82 ± 0.13	78.30 ± 0.16	84.55 ± 0.16
S11	70.74 ± 0.05	54.40 ± 0.25	42.45 ± 0.12	64.04 ± 0.27	75.18 ± 0.10
S12	95.80 ± 0.07	50.72 ± 0.22	52.69 ± 0.01	70.31 ± 0.31	89.33 ± 0.12
S13	63.75 ± 0.09	34.45 ± 0.14	33.17 ± 0.3	61.58 ± 0.25	69.52 ± 0.26
S14	30.12 ± 0.05	54.24 ± 0.22	40.05 ± 0.02	60.74 ± 0.26	60.88 ± 0.27
S15	86.15 ± 0.07	47.25 ± 0.38	51.05 ± 0.02	76.10 ± 0.37	86.24 ± 0.14
S16	95.69 ± 0.06	90.21 ± 0.31	61.73 ± 0.08	83.67 ± 0.14	90.10 ± 0.03
S17	107.27 ± 0.07	97.28 ± 0.14	65.17 ± 0.09	83.98 ± 0.04	91.88 ± 0.09
S18	103.81 ± 0.14	71.80 ± 0.48	55.96 ± 0.05	76.07 ± 0.16	89.13 ± 0.16
S19	90.73 ± 0.07	65.90 ± 0.25	51.77 ± 0.08	78.32 ± 0.04	90.78 ± 0.15
S20	72.85 ± 0.03	53.98 ± 0.10	40.27 ± 0.05	62.28 ± 0.06	73.93 ± 0.08
S21	61.50 ± 0.04	50.67 ± 0.17	43.64 ± 0.01	63.22 ± 0.03	67.39 ± 0.11
S22	95.36 ± 0.14	82.89 ± 0.37	53.29 ± 0.13	83.06 ± 0.12	87.83 ± 0.06
S23	91.90 ± 0.04	51.87 ± 0.22	46.67 ± 0.02	76.11 ± 0.14	85.29 ± 0.04
Statistical difference	**	**	**	**	**

** Statistical difference was expressed based on one-way ANOVA test (*P* < 0.01).

### LC-ECD fingerprinting

**Fig. 1:**
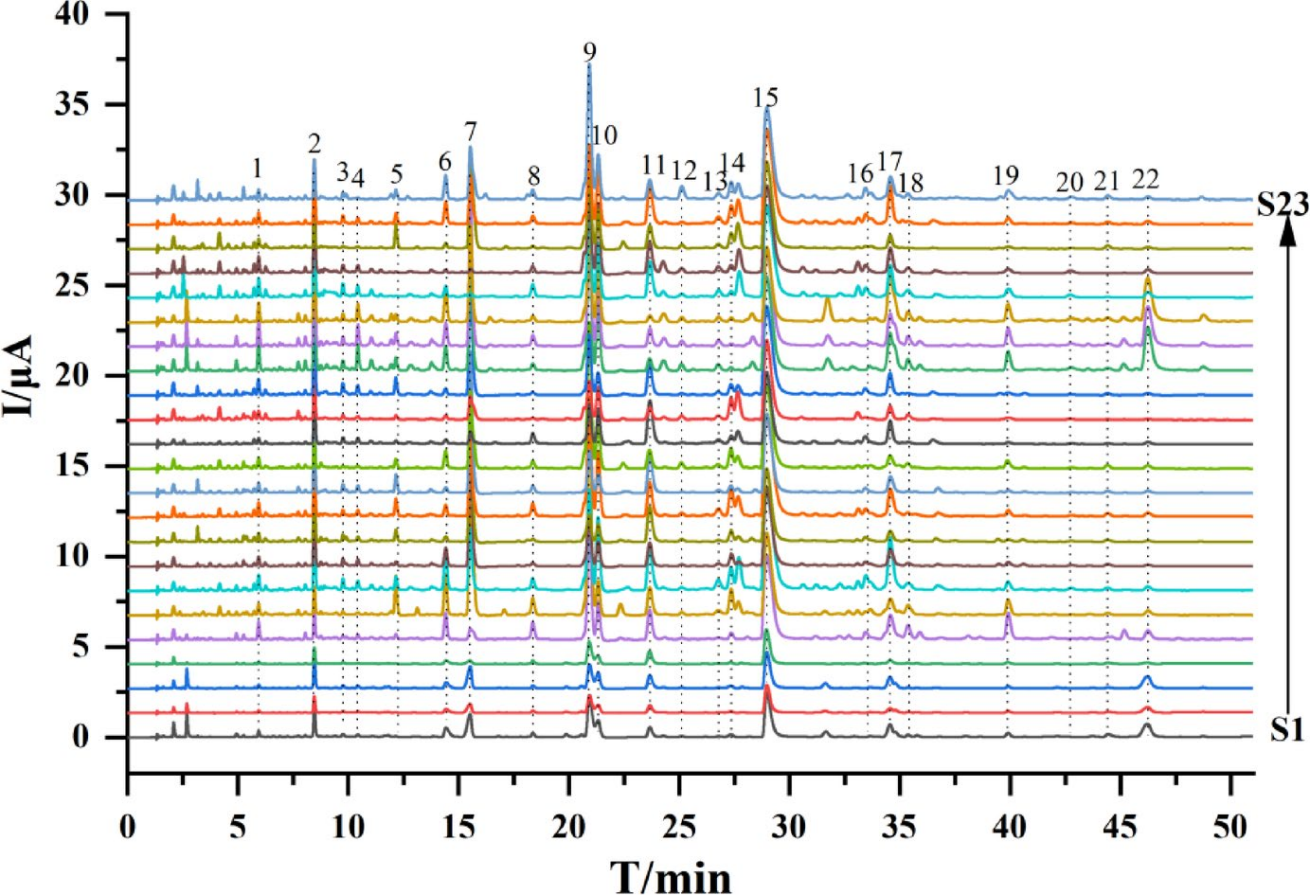
LC-ECD fingerprints for twenty-three batches of sweet tea. Numbers indicate compounds: (1) Protocatechuic acid; (2) Catechin; (3) Epicatechin; (4) Phloretin-dihexoside; (5) (E)-4- (3,4-Dihydroxyphenyl)-N-(1-hydroxy-2-(4-hydroxyphenyl)-ethyl)-2-oxobutyl-3-enamide; (6) Phloretin-dihexoside; (7) 3-Hydroxyphlorizin isomer (8) *p*-Coumaric acid; (9) 3-Hydroxyphlorizin; (10) Isoquercitrin; (11) Phlorizin; (12) Phloretin-acetyl-hexoside; (13) Cynaroside isomer; (14) Quercitrin; (15) Trilobatin; (16) Phloretin-malonyl-hexoside; (17) Phloretin-dihydroxyphloretin-hexoside; (18/19) Phloretin-dihydroxyphloretin-dihexoside; (20) Quercetin; (21) Phloretin-acetyl-hexoside; (22) Phloretin.Identification of the compounds.

LC-ECD is capable of specifically detecting antioxidant components, particularly phenolic compounds^37^. Twenty-three batches of sweet tea were prepared and analyzed using LC-ECD. After multi-point correction, the chromatograms of the samples were matched to obtain the fingerprints as described previously^38^. A total of 22 peaks were present in all the samples, i.e. common peaks labeled P1-P22 in the chromatograms, as shown in Fig. 1. Compounds corresponding to peaks with larger peak areas, such as P7, P9, P10, and P15, are anticipated to have considerable reducing power because ECD detects charge transfer in electrochemical reactions.

**Fig. 2:**
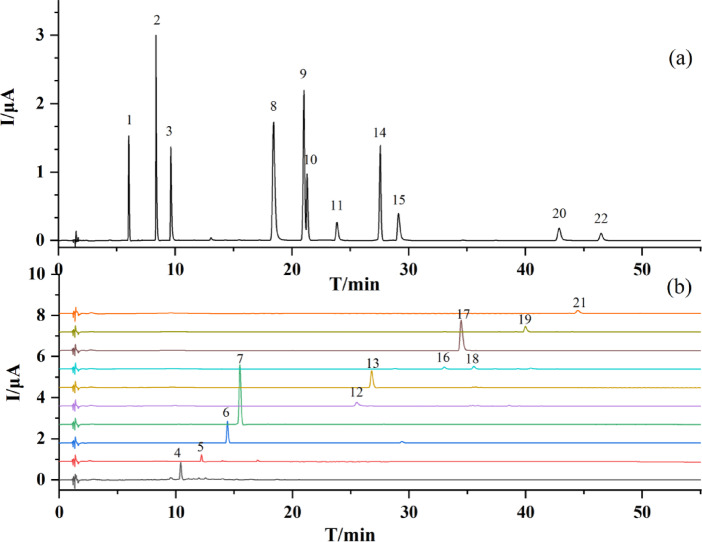
Chromatogram of (a) the standards and (b) different fractions. Numbers 1–22 represent 22 common peaks.

**Fig. 3:**
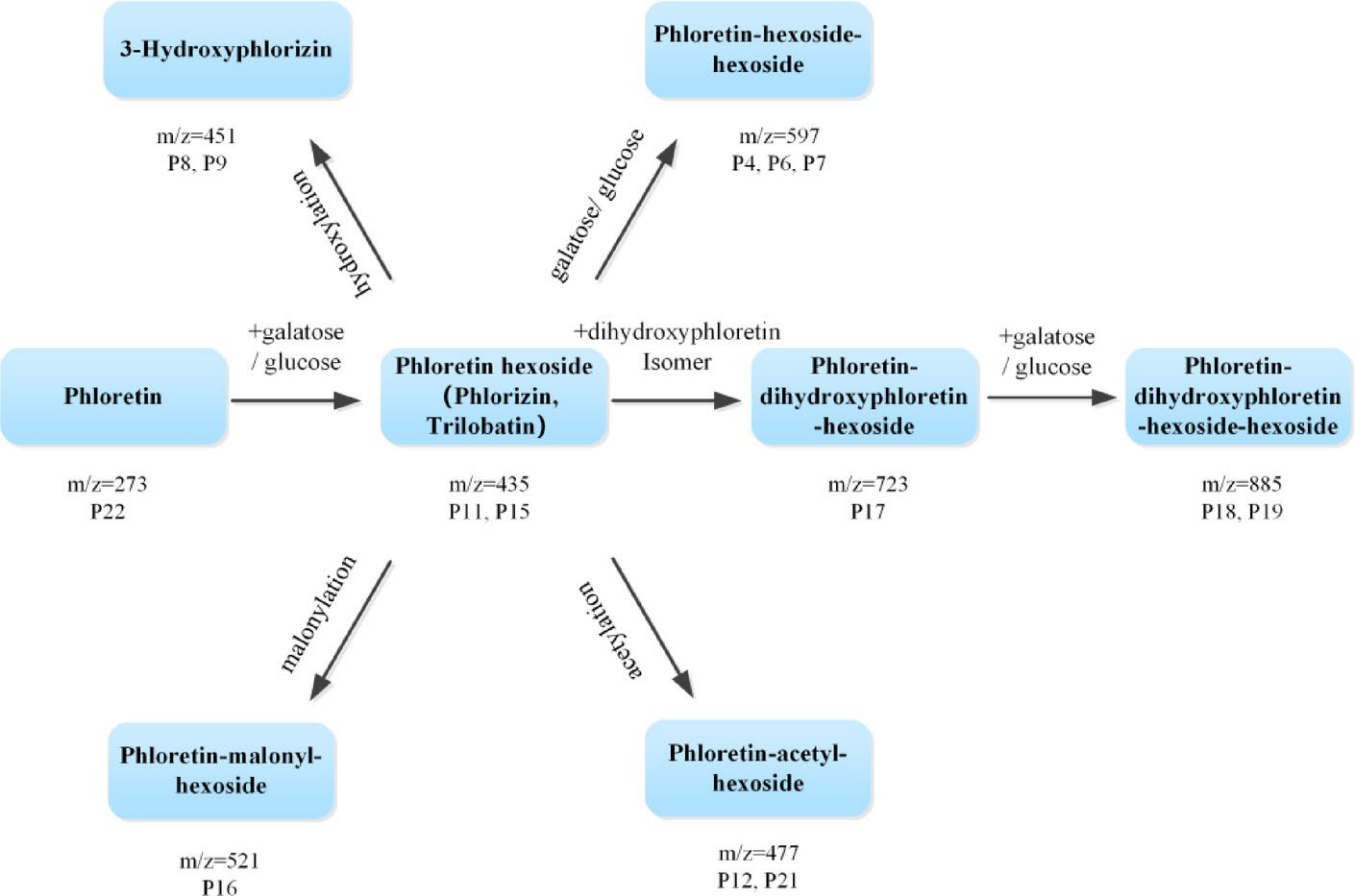
Major phloretin derivatives in sweet tea.

The major peaks isolated and collected by semi-preparative chromatography at 280 nm were identified by LC-MS/MS. Scanning was performed in negative mode, and the product ions were analyzed. As a result, precursor ions and fragment ions of the 22 common peaks were obtained, and the results are presented in Table 3. The fractions containing the identified compounds were then injected into LC-ECD to confirm the common peaks. Some of them were verified using standard substances, including protocatechuic acid, catechin, epicatechin, 3-hydroxyphlorizin, *p*-coumaric acid, isoquercitrin, phlorizin, quercitrin, trilobatin, quercetin, and phloretin. The chromatogram of the standards is shown in Fig. 2a, and the overlapped chromatograms of different fractions are shown in Fig. 2b. It can be observed that the compounds with the highest quantity and the largest proportion in sweet tea are phloretin and its derivatives. The representative compounds are the conjugates of phloretin and glucose, such as phlorizin and trilobatin. Phloretin-hexoside can further bind other groups, undergo acetylation, hydroxylation, etc. to form other complex compounds, as illustrated in Fig. 3. In the mass spectra fragments of these compounds, the product ion at m/z 273 is commonly present, which corresponds to the ionization product of phloretin.

**Table 3:** LC-MS/MS identification of the phenolic compounds in sweet tea.

Number	Precursor ion (m/z)	Product ions (m/z)	Identification
1	153	135, 109	Protocatechuic acid
2	289	125, 245, 179, 165, 151	Catechin
3	289	245, 203, 123, 109, 161	Epicatechin
4	597	435, 273	Phloretin-dihexoside
5	342	135, 161, 119, 163, 180, 206, 101, 133	(E)-4- (3,4-Dihydroxyphenyl)-N- (1-hydroxy-2- (4-hydroxyphenyl)-ethyl)-2-oxobutyl-3-enamide
6	597	435, 273	Phloretin- dihexoside
7	451	167, 125, 289, 271, 245	3-Hydroxyphlorizin isomer
8	163	119, 145	*p*-Coumaric acid
9	451	167, 289, 191, 125, 271, 209, 178, 271	3-Hydroxyphlorizin
10	463	301, 300, 151, 178, 271	Isoquercitrin
11	435	273, 167, 125, 178	Phlorizin
12	477	273, 167, 297, 339	Phloretin-acetyl-hexoside
13	447	284, 285, 255, 151, 227, 327	Cynaroside isomer
14	447	300, 301, 151, 179, 255, 271	Quercitrin
15	435	273, 167, 191, 209, 297, 125, 179, 201, 315	Trilobatin
16	521	273, 297, 167, 191, 315	Phloretin-malonyl-hexoside
17	723	391, 125, 273, 287, 435	Phloretin-dihydroxyphloretin-hexoside
18	885	165, 149, 194, 361, 509, 329, 107, 321, 385, 477, 273	Phloretin-dihydroxyphloretin-dihexoside
19	885	723, 561, 393, 435, 725, 175, 341	Phloretin-dihydroxyphloretin-dihexoside
20	301	179,151, 145	Quercetin
21	477	351, 161, 101, 273, 125	Phloretin-acetyl-hexoside
22	273	123, 167, 119, 125, 151, 189	Phloretin

Currently, there have been numerous studies on the chemical composition of sweet tea, and with the help of liquid chromatography-high resolution mass spectrometry, the main sources of sweetness in sweet tea, such as phloretin and 3-hydroxyphloretin derivatives, represented by their glycosides like trilobatin and phlorizin, have been identified^7^. In addition, there are also complex compounds such as acetyl-phlorizin and galloyl-phlorizin that possess phenolic structures and are also the main sources of sweet tea’s antioxidant activity^19^. However, previous studies mainly focused on identifying chemical components in sweet tea and failed to clarify the contribution of different compounds to antioxidant activity. Meanwhile, the response of mass spectrometry is influenced by various factors, making it difficult to accurately represent the contents of different compounds. Consequently, the relative contents of phenolic compounds in sweet tea from different sources have not been fully investigated.

LC-ECD selectively targets phenolic hydroxyl groups via charge transfer, offering superior specificity for antioxidant detection compared to UV-based methods. A large number of studies have confirmed that ECD has a wide range of applications in the evaluation of antioxidant activity^38^. When combined with HPLC separation, it can also specifically screen for antioxidant components. The phenolics and flavonoids of sweet tea are abundant, and most of them have one or more phenolic hydroxyl structures. Therefore, the fingerprinting study using LC-ECD can provide a comprehensive profile of the antioxidant components of phenolic compounds. The role of other potential antioxidant components, such as polysaccharides, terpenoids, and vitamin C in sweet tea has not been systematically evaluated.

### Cluster heat map analysis

**Fig. 4:**
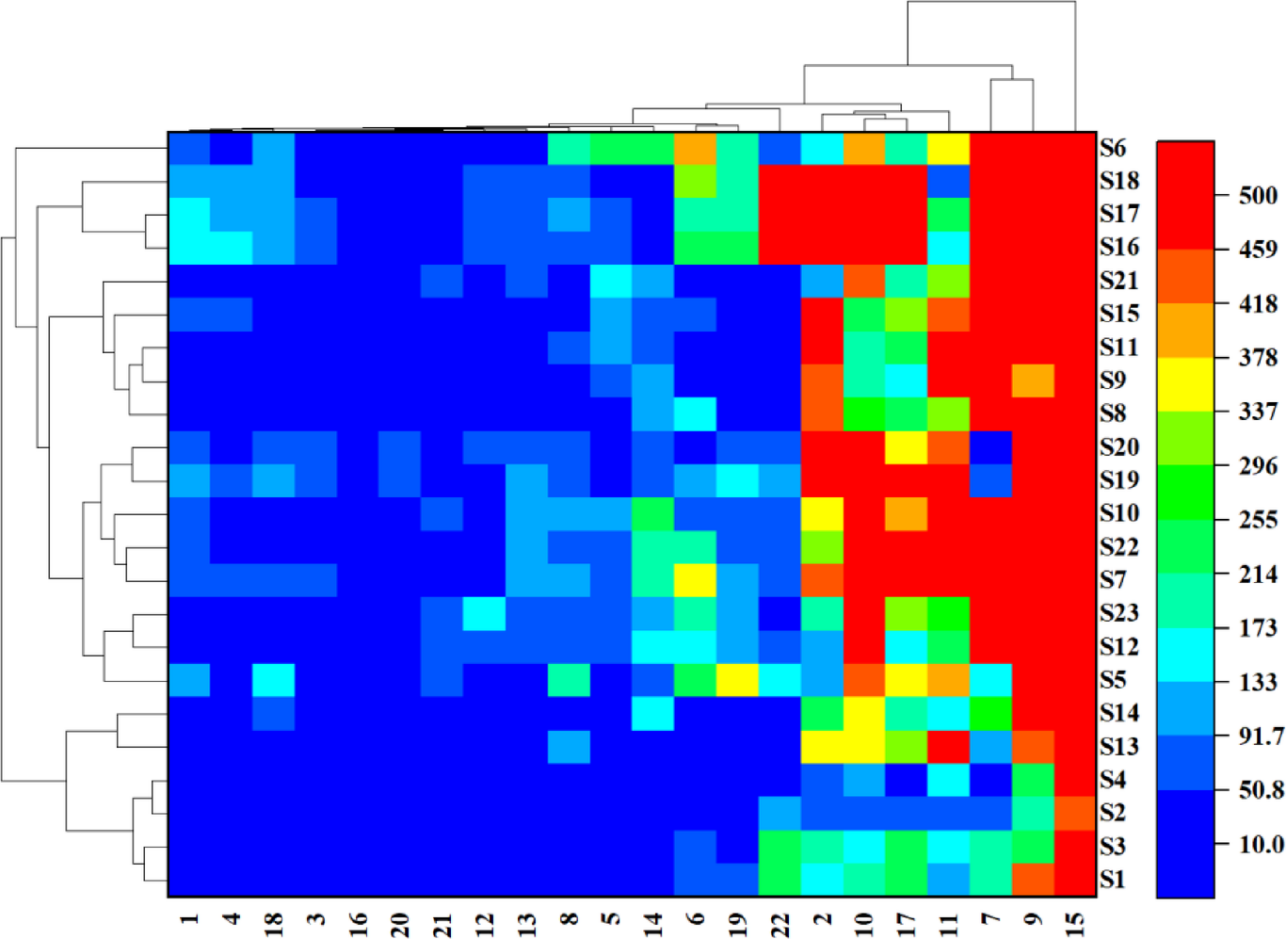
Squared Euclidean distances were used to analysis the samples and peaks in a clustered heat map. The horizontal coordinates represent each of the 22 shared peaks and the vertical coordinates represent the 23 batches of samples. The blue-to-red color gradient visually maps increasing peak areas.

To explore the differences in compounds between different samples, 23 batches of samples were classified and analyzed. Heat map analysis was carried out to visualize the relative content of compounds, with color variations indicating differences in content ranging from low (blue) to high (red)^39,40^. The results are shown in Fig. 4. The 22 common peaks can be categorized into three groups. Trilobatin was clustered into one category by itself, 3-hydroxyphlorizin and its isomer (P7) were clustered into one category, and the remaining compounds were clustered into one category. Since the peak area of trilobatin was the largest in sweet tea, followed by 3-hydroxyphlorizin and its isomer with larger peak areas, they may be the main components in sweet tea. According to literature reports, sweet tea primarily contains dihydrochalcones, including 3-hydroxyphlorizin, phlorizin, trilobatin, etc^4,21^. As major components in sweet tea, previous pharmacological studies have demonstrated their significant roles in antioxidant activity. These components have been shown to alleviate oxidative stress damage via the Nrf2/ARE signaling pathway^41,42^.

There were also significant differences in the samples from different sources. When the Euclidean distance is 1500, the 23 batches of samples can be divided into four categories. Among them, S1, S2, S3, S4, S13, and S14 from Yunnan and Jiangxi (Shangrao and Pingxiang) were categorized as the first group. Then, S5, S7, S8, S9, S10, S11, S12, S15, S19, S20, S21, S22, and S23, mainly from Sichuan, Hunan, Guangxi, and Jiangxi (Jian and Pingxiang), were categorized into the second group. The rest of the samples S16, S17, and S18 from Guizhou were in the third group. S6 (Anhui) was the fourth group. Due to the wide range of sample origins, samples from different regions have certain overlaps in the clustering analysis; S14 and S19 clustering crosses over with other regions. This divergence may arise from biological factors during plant growth^43^, such as genetic variation of endogenous microbial activity or metabolic pathways. However, abiotic factors, including microclimate differences (such as annual average precipitation and light intensity) and soil differences (such as pH, nitrogen, and phosphorus levels), in different production areas have complex interactions on the biosynthesis of secondary metabolites in plant metabolism^44^. The samples from Guizhou (S16-S18) contain high phenolic and flavonoid components, which may be due to the unique geographical and climatic environment. The calcium-rich karst landform in Guizhou may promote the biosynthesis of flavonoids because it is well known that calcium signaling can regulate the phenylpropane pathway^45^. Studies have also shown that high altitude (above 1500 m) is conducive to the accumulation of phenolic compounds^46^. In contrast, the lower content of compounds in Yunnan samples (S1-S4) may reflect factors related to temperature and soil acidity.

Sampling was carried out in six provinces in southern China. However, the sampling distribution across the provinces was uneven. For example, Guizhou had only two batches, while Jiangxi had eight batches. This uneven sampling might affect the regional representativeness. In future studies, the sampling scope will be expanded to regions with climates similar to those in China, such as Vietnam and India, to enhance the universality of the research results. Additionally, controlled cultivation experiments will be incorporated to distinguish the effects of environmental factors from genetic variations.

### Correlation analysis

To verify the reliability of LC-ECD in screening antioxidant compounds, a correlation analysis was conducted between the total peak area in LC-ECD chromatograms (Fig. 1) and TPC, TFC, DPPH radical scavenging capacity, ABTS radical scavenging capacity, and FRAP value (see Table 4). The correlation coefficients were calculated, resulting in values of 0.918, 0.924, 0.934, 0.927, and 0.929, respectively.

**Table 4:** Pearson’s correlation coefficients between total peak area of samples, TFC, TPC and in vitro antioxidant activities.

	Total peak area	TPC	TFC	DPPH	ABTS	FRAP
Total peak area	1					
TPC	0.918**	1				
TFC	0.924**	0.817**	1			
DPPH	0.934**	0.934**	0.896**	1		
ABTS	0.927**	0.970**	0.832**	0.951**	1	
FRAP	0.929**	0.855**	0.892**	0.864**	0.868**	1

**Indicates significant correlation (*P* < 0.01).

**Fig. 5:**
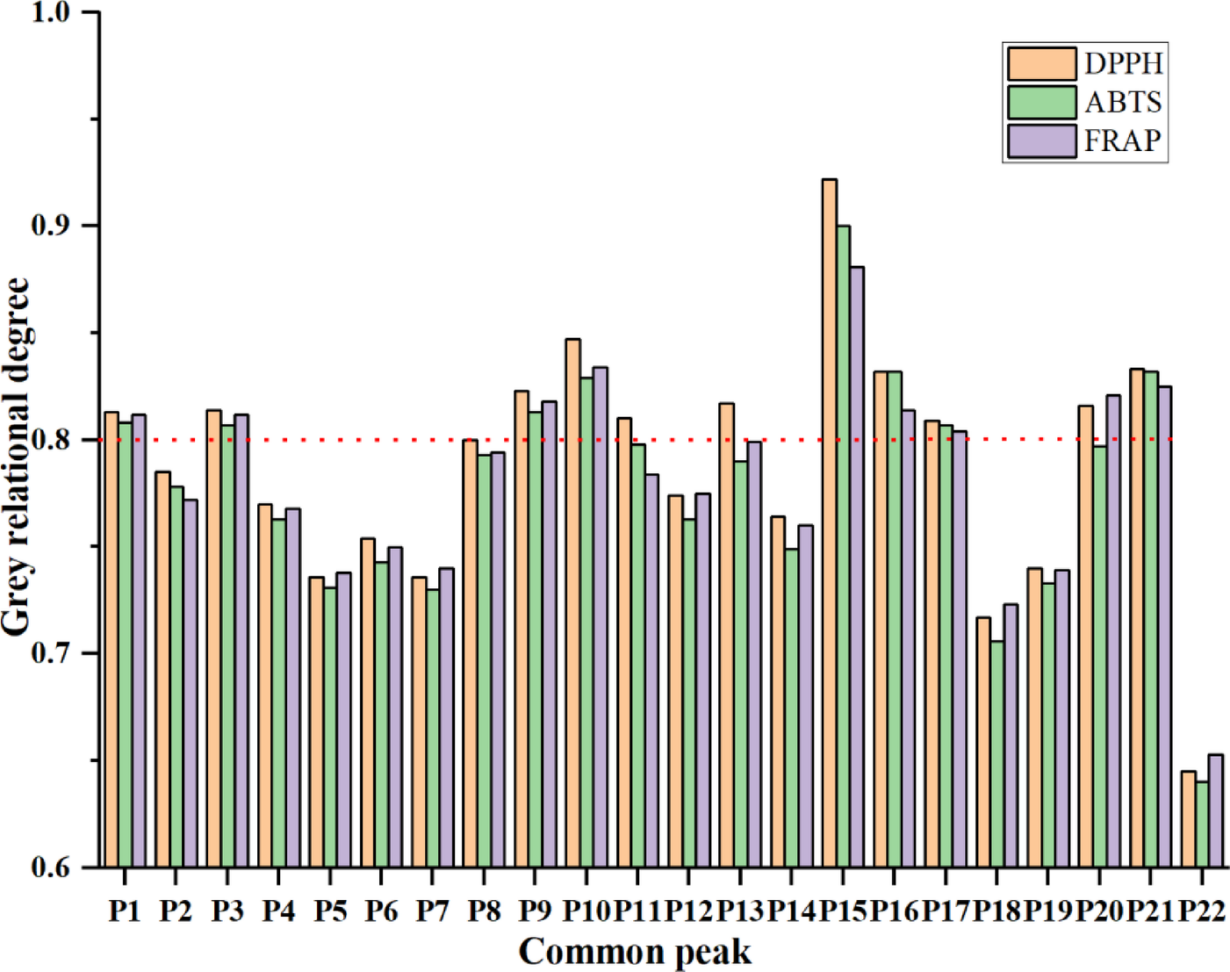
Gray correlation plots of 22 common peaks of sweet tea with in vitro antioxidant activity. The P1 ~ P22 serial numbers indicate the common peaks in the fingerprints.

To further clarify the correlation between different compounds and antioxidant activity, GRA was performed. GRA, as a multivariate method, evaluates the nonlinear relationships among variables by comparing the similarities of data sequences^47^. It quantifies the correlations between chromatographic peaks and pharmacological indicators, such as antioxidant activity, to identify key bioactive components^48^. The gray relational degree (GRD) value indicates the strength of the correlation in antioxidant activities; larger values imply more significant correlations^49^. In this study, correlation analysis was conducted by measuring 22 peak areas and antioxidant activity data (ABTS, DPPH, FRAP). The results are shown in Fig. 5. The correlation degrees between the 22 common peaks and antioxidant activities were all greater than 0.6. Among them, the chromatographic peaks are ranked in descending order of their contribution to antioxidants (GRD > 0.8) as follows: 15 (trilobatin), P10 (isoquercitrin), P21 (phloretin-acetyl-hexoside), P16 (phloretin-malonyl-hexoside), P9 (3-hydroxy phlorizin), P1 (protocatechuic acid), P3 (epicatechin), and P17 (phloretin-dihydroxyphloretin-hexoside), suggesting that these eight components play a key role. The highest correlation was found for peak P15 with an average correlation degree greater than 0.9, indicating a close association with antioxidant activity. Inspired by the work of Gao et al., who applied GRA to screen antioxidant components in red wine^50^.Our GRA results align with recent studies on plant antioxidants. Studies have shown that in the universal efficacy analysis of Rhizoma Paridis extracts, components with GRD>0.75 can be considered key active substances^51^. Recent studies report that dihydrochalcones in sweet tea, such as trilobatin and phlorizin, function as potent antioxidant-active compounds^4,21^. These compounds exhibit antidiabetic effects by modulating blood glucose/lipid metabolism, mitigating oxidative/carbonyl stress, and suppressing inflammatory responses^52^. Notably, the GRD value of trilobatin exceeded 0.9 in our experiments, underscoring its pivotal contribution to the antioxidant efficacy of sweet tea. These findings validate the reliability of HPLC-ECD for targeted screening of antioxidant components. Future research should focus on two directions: (1) evaluating the impact of processing techniques (e.g., drying, fermentation) on the retention of bioactive compounds, and (2) conducting in vivo studies to confirm the therapeutic potential of identified molecules, particularly trilobatin, in diabetes management.

**Fig. 6:**
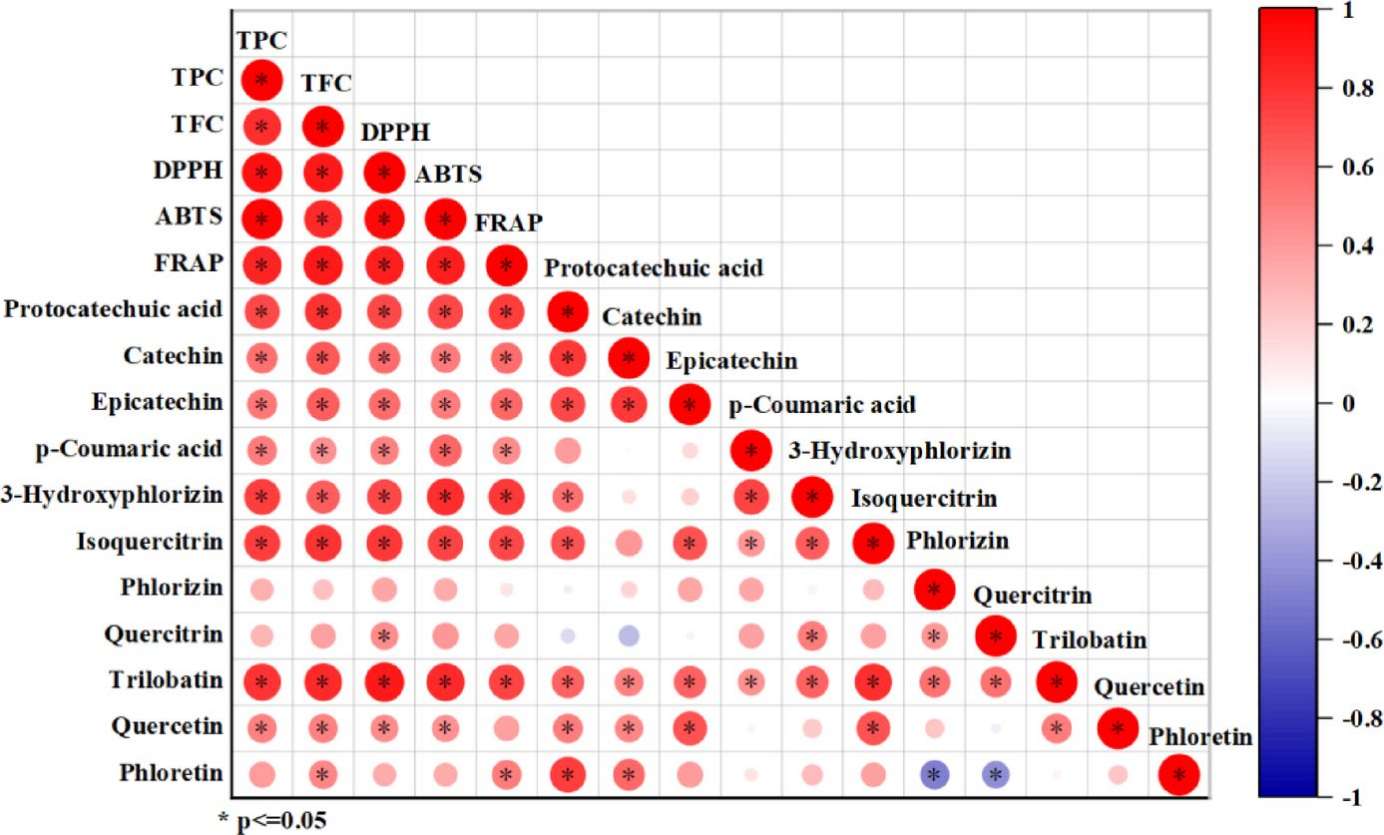
Pearson’s correlation coefficients of 11 components of sweet tea with TFC and TPC and antioxidant activity.

Pearson’s correlation coefficient is a statistical indicator that measures the strength and direction of linear relationships between two continuous variables. For instance, it has been used to disclose the association between the contents of active components in *Gnaphalium affine* and antioxidant activity indices^34^. In this study, Pearson’s correlation analysis was performed between the 11 compounds quantified after identification and the results of TPC, TFC, and antioxidant activity, as depicted in Fig. 6. Protocatechuic acid, catechin, epicatechin, *p*-coumaric acid, 3-hydroxyphlorizin, isoquercitrin, trilobatin, and quercetin were found to have a high degree of interdependence with antioxidant activity, while other compounds also exhibited correlations. The literature indicates that synergistic interactions among multiple components in plant extracts can prolong free-radical scavenging chain reactions through cooperative electron transfer^53^. This study further demonstrated that compound combinations with high correlations may enhance antioxidant efficacy via such synergistic mechanisms^28^.

The results of Pearson’s correlation analysis and GRA were highly consistent with online screening results from ECD, indicating stronger links between chemical substances with larger peak areas and antioxidant activity. Compared to traditional isolation methods, this approach efficiently identifies key antioxidants while clarifying synergistic effects, offering a robust framework for rapid bioactive compound screening in plant extracts.

### Determination of 11 phenolic compounds in sweet tea from different origins

The linear regression equations, correlation coefficients (*R*²), and recovery rates were calculated. The results are presented in Table S1 of the Supplementary Information. All 11 phenolic compounds demonstrated good linearity within their respective concentration ranges, with *R*² exceeding 0.99. The detection limits, calculated using a signal-to-noise ratio of 3, ranged from 2.76 to 8.98 µg/L. Additionally, recovery rates ranged from 93.68 to 105.54%, indicating good accuracy of the method.

**Fig. 7:**
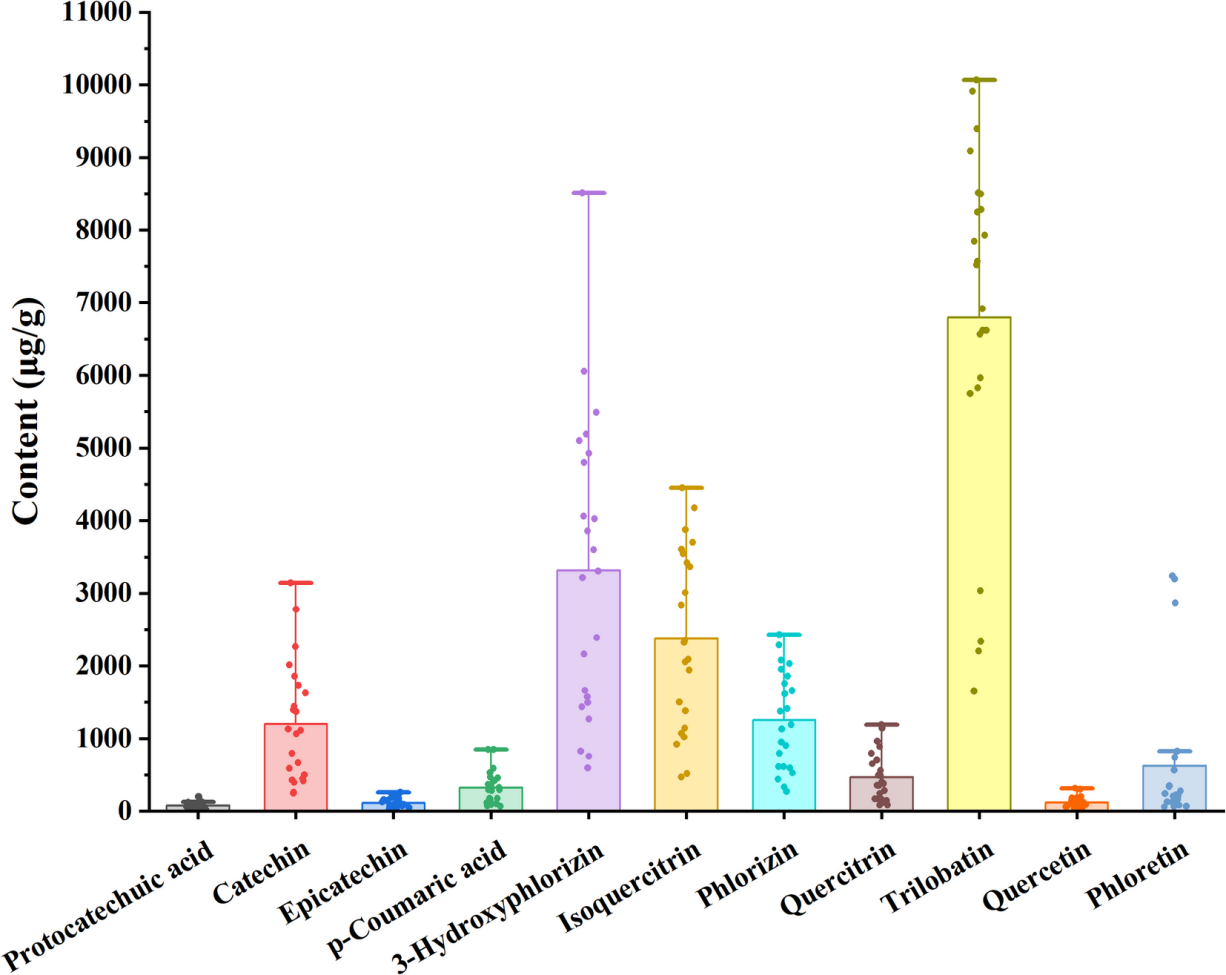
Content of 11phenolic compounds in different batches of sweet tea. Scattered dots indicate the distribution of the compounds in different samples.

The content of the 11 phenolic compounds in 23 batches of sweet tea from different origins was determined in triplicate, and the results are shown in Table 5; Fig. 7. Table 5 presents the mean values with SD of the compound contents, while Fig. 7 illustrates the distribution of constituent contents across samples. The average content of trilobatin was the highest, followed by 3-hydroxyphlorizin, isoquercitrin, phlorizin, catechin, and phloretin. The emphasis on average content highlights the predominant compounds contributing to the overall phenolic profile, which is critical for evaluating the bioactive potential and regional characteristics of sweet tea.

**Table 5:** Determination of 11 phenolic compounds in 23 batches of samples (µg /g). Data are reported as mean ± sd (*n* = 3).

	Protocatechuic acid	Catechin	Epicatechin	*p*-Coumaric acid	3-Hydroxyphlorizin	Isoquercitrin	Phlorizin	Quercitrin	Trilobatin	Quercetin	Phloretin
S1	43.8 ± 0.88	498.0 ± 7.47	69.2 ± 0.90	174.6 ± 2.32	1437.3 ± 16.31	1019.2 ± 17.53	439.6 ± 7.13	171.0 ± 2.94	3032.5 ± 36.39	47.3 ± 0.58	821.9 ± 15.62
S2	14.9 ± 0.31	256.8 ± 5.39	29.5 ± 0.32	90.4 ± 1.29	593.7 ± 8.52	469.9 ± 7.61	332.1 ± 4.06	83.2 ± 1.35	1654.2 ± 18.20	47.9 ± 0.54	348.4 ± 4.53
S3	23.8 ± 0.38	590.9 ± 7.11	58.3 ± 0.82	117.2 ± 1.56	827.0 ± 17.66	921.3 ± 15.58	613.8 ± 10.58	166.8 ± 2.87	2338.8 ± 37.42	59.0 ± 0.78	742.1 ± 8.91
S4	16.5 ± 0.31	247.3 ± 5.69	37.1 ± 0.45	154.5 ± 2.36	755.2 ± 16.88	518.9 ± 8.93	613.6 ± 10.57	85.6 ± 1.47	2203.7 ± 41.87	41.6 ± 0.47	82.4 ± 1.15
S5	121.9 ± 2.44	427.4 ± 8.89	43.9 ± 0.48	850.5 ± 8.73	4925.1 ± 49.25	2326.3 ± 40.01	1410.9 ± 17.26	356.5 ± 6.13	7568.7 ± 83.26	59.6 ± 0.85	566.7 ± 6.23
S6	67.1 ± 1.14	449.4 ± 4.94	52.3 ± 0.68	845.5 ± 8.68	8512.4 ± 86.83	2091.3 ± 35.97	1374.7 ± 15.44	1141.9 ± 19.64	6915.8 ± 78.15	30.4 ± 0.52	279.9 ± 4.76
S7	95.7 ± 1.72	1397.1 ± 15.58	241.8 ± 2.90	456.1 ± 6.05	3858.2 ± 49.22	4449.4 ± 54.28	2290.6 ± 29.78	886.5 ± 10.82	10065.7 ± 143.90	147.1 ± 1.19	215.7 ± 4.10
S8	57.8 ± 1.12	1444.7 ± 17.34	82.2 ± 1.15	104.5 ± 1.39	2163.3 ± 86.83	1501.7 ± 25.83	1128.7 ± 13.54	561.9 ± 9.66	6617.3 ± 88.01	96.8 ± 1.45	62.7 ± 1.44
S9	53.9 ± 1.46	1369.3 ± 17.11	70.7 ± 0.92	76.1 ± 1.01	1269.6 ± 59.22	1070.0 ± 20.54	1854.6 ± 25.96	492.5 ± 9.46	5964.2 ± 101.11	111.3 ± 1.34	65.1 ± 1.89
S10	84.2 ± 1.68	1113.7 ± 25.62	127.5 ± 1.14	592.6 ± 5.33	4798.7 ± 28.88	3874.8 ± 43.40	1755.3 ± 33.35	1191.4 ± 13.34	9911.2 ± 138.76	83.1 ± 1.16	242.8 ± 8.01
S11	59.1 ± 1.18	2010.2 ± 26.13	110.4 ± 1.66	370.1 ± 4.91	1662.5 ± 27.11	1141.1 ± 19.63	2031.0 ± 32.96	282.1 ± 4.85	6566.6 ± 80.11	93.2 ± 0.95	124.0 ± 3.60
S12	60.3 ± 1.03	393.9 ± 9.06	24.1 ± 0.31	357.2 ± 5.10	6052.7 ± 81.58	3005.9 ± 51.70	900.4 ± 12.81	792.7 ± 10.46	7519.8 ± 92.49	82.8 ± 1.26	208.2 ± 2.50
S13	33.5 ± 0.70	1131.0 ± 13.57	97.4 ± 1.27	470.4 ± 6.24	1499.1 ± 35.49	2056.2 ± 35.37	2428.6 ± 39.34	243.9 ± 3.71	5747.0 ± 81.61	63.8 ± 0.84	126.5 ± 1.90
S14	52.7 ± 0.84	795.0 ± 12.72	91.7 ± 1.19	65.5 ± 0.87	1577.3 ± 66.58	1941.0 ± 33.39	594.2 ± 6.67	701.7 ± 9.26	6616.6 ± 82.05	70.6 ± 0.86	57.7 ± 0.98
S15	95.0 ± 1.24	2264.3 ± 31.96	161.2 ± 2.10	174.7 ± 2.14	2388.9 ± 32.01	1383.7 ± 23.80	1616.5 ± 19.77	403.6 ± 5.73	8282.6 ± 91.11	72.5 ± 0.89	134.5 ± 2.96
S16	201.3 ± 2.01	3143.0 ± 31.43	214.3 ± 3.46	326.2 ± 4.16	5100.2 ± 33.68	3547.3 ± 46.82	526.4 ± 8.53	146.3 ± 1.93	8495.7 ± 84.96	162.3 ± 3.12	3198.0 ± 51.21
S17	196.6 ± 2.56	2778.1 ± 36.12	191.0 ± 2.48	530.1 ± 7.03	5190.3 ± 51.00	3603.8 ± 51.17	791.3 ± 12.63	112.0 ± 1.59	8513.9 ± 87.69	169.0 ± 3.25	2867.5 ± 37.31
S18	127.7 ± 1.66	1728.6 ± 39.76	159.1 ± 2.55	291.5 ± 4.16	4025.5 ± 56.89	3699.9 ± 48.84	272.8 ± 3.34	171.0 ± 2.26	5827.1 ± 75.75	180.5 ± 4.01	3236.8 ± 38.87
S19	128.9 ± 2.58	1855.5 ± 38.91	257.1 ± 3.34	422.6 ± 5.61	4064.1 ± 81.28	3368.2 ± 57.93	1952.0 ± 33.63	352.2 ± 6.06	9393.8 ± 103.00	315.5 ± 8.59	336.0 ± 4.70
S20	105.3 ± 2.00	1629.9 ± 37.49	178.2 ± 2.32	280.5 ± 3.72	3601.0 ± 76.88	4176.2 ± 50.95	1659.0 ± 18.63	378.8 ± 4.62	8248.0 ± 97.02	299.0 ± 3.90	224.4 ± 3.81
S21	47.5 ± 0.93	415.0 ± 8.30	59.8 ± 0.66	102.9 ± 1.16	3307.0 ± 44.15	2347.1 ± 40.37	1189.1 ± 16.92	492.3 ± 5.51	7844.8 ± 101.31	123.8 ± 3.00	112.6 ± 2.03
S22	64.5 ± 1.48	1064.9 ± 14.91	123.0 ± 1.66	331.4 ± 4.40	3213.7 ± 49.33	3419.1 ± 40.07	2077.4 ± 31.64	961.6 ± 11.27	9086.4 ± 99.95	201.0 ± 5.47	212.9 ± 4.05
S23	59.0 ± 1.18	666.4 ± 8.66	72.8 ± 0.95	295.6 ± 3.63	5491.5 ± 61.50	2831.9 ± 34.55	952.4 ± 12.60	652.5 ± 7.96	7929.1 ± 93.11	183.5 ± 3.35	163.2 ± 3.75

The contents of quercetin, *p*-coumaric acid, epicatechin, and protocatechuic acid were relatively low. Additionally, the total content of 11 phenolic compounds was higher in samples S17 and S16, both from Guizhou Province, indicating their better quality. The observed geographical correlation suggests that environmental factors in Guizhou, such as soil composition and climate, may promote the biosynthesis of phenolic compounds. Generally, a higher phenolic content is associated with stronger antioxidant capacity and more health benefits. Thus, it can be inferred that samples S16 and S17 possess superior functional quality. Given this, the unique terroir conditions of Guizhou could be given priority for commercial cultivation in order to maximize the production of antioxidants.

In future research directions, first, efforts should be focused on the dynamic accumulation patterns of phenolic substances. By combining correlation analysis with environmental factors, the seasonal fluctuation characteristics and time-dependent control mechanisms can be revealed, thus improving the theoretical framework for resource development. Then, the research scope should be extended to the validation of transformation. A type-2 diabetes mouse model can be used to conduct in vivo efficacy evaluation. Synchronously, pharmacokinetic analysis and the detection of antioxidant enzyme activities in liver and kidney tissues should be integrated to systematically elucidate the bioavailability, metabolic trajectory, and therapeutic potential of antioxidant components, thereby promoting the transition from basic research to clinical application.

## Conclusion

This study established LC-ECD coupled with LC-MS/MS as a robust platform for the rapid screening of sweet tea antioxidants, filling a critical gap in the quality control of this understudied plant. We determined that trilobatin is an important contributor to the antioxidant properties of sweet tea. Our geographical clustering model provides actionable insights for sourcing high-quality sweet tea, with samples from Guizhou showing superior phenolic content. This work advances phytochemical analysis by demonstrating how the integration of dual technology accelerates the discovery of antioxidants, offering a blueprint for the standardization of natural products. Future efforts should focus on addressing regional harvesting practices and exploring how environmental factors shape antioxidant profiles.

## Electronic supplementary material

Below is the link to the electronic supplementary material.

Supplementary Material 1

## Data Availability

All data generated or analysed during this study are included in this article and its Supplementary Information files.
