# Supplementary material for: Antioxidant profiling and quality assessment of Lithocarpus polystachyus sweet tea using LC-ECD and LC-MS/MS
Source: Sci Rep. 2025 Apr 16;15:13163. doi: 10.1038/s41598-025-97875-7 (PMC12003848; doi:10.1038/s41598-025-97875-7)
Supplement: Supplementary file 1 — Supplementary Material 1 [file 41598_2025_97875_MOESM1_ESM.docx]

**Table S1.** Linearity and Recovery of 11 phenolic compounds.

| **Compounds** | **Linear equation** | **Correlation coefficient**  **（R^2^）** | **Linear range**  **(mg/L)** | **Limit of detection**  **(μg/L)** | **Average recovery**  **(%)** | **RSD**  **(%)** |
| --- | --- | --- | --- | --- | --- | --- |
| Protocatechuic acid | y = 24.828x + 0.4063 | 0.9995 | 0.4~40 | 2.81 | 102.65 | 6.36 |
| Catechin | y = 9.8022x + 0.5720 | 0.9953 | 1.5~150 | 3.86 | 99.34 | 4.12 |
| Epicatechin | y = 5.1559x - 2.5686 | 0.9980 | 0.4~40 | 5.98 | 93.78 | 4.72 |
| *p*-Coumaric acid | y = 6.2048x - 1.7088 | 0.9992 | 0.4~40 | 3.55 | 95.40 | 3.62 |
| 3-Hydroxyphlorizin | y = 9.1629x - 3.4778 | 0.9999 | 3~300 | 3.65 | 99.73 | 4.72 |
| Isoquercitrin | y = 5.4711x - 0.8379 | 1.0000 | 2~200 | 8.98 | 103.56 | 3.92 |
| Phlorizin | y = 8.2368x - 7.8314 | 0.9936 | 1~100 | 2.76 | 105.54 | 5.18 |
| Quercitrin | y = 5.8775x - 0.9481 | 0.9999 | 1~100 | 7.53 | 99.89 | 5.24 |
| Trilobatin | y = 8.2544x - 7.7671 | 0.9953 | 4~400 | 3.87 | 100.21 | 2.72 |
| Quercetin | y = 6.0374x - 0.1139 | 0.9999 | 0.4~40 | 6.38 | 93.68 | 5.02 |
| Phloretin | y = 8.9682x - 6.5181 | 0.9932 | 1.5~150 | 6.99 | 98.25 | 5.32 |
